# Outlier detection of clustered functional data with image and signal processing applications by archetype analysis

**DOI:** 10.1371/journal.pone.0311418

**Published:** 2024-11-25

**Authors:** Aleix Alcacer, Irene Epifanio

**Affiliations:** 1 Department of Mathematics, Universitat Jaume I, Castelló, Spain; 2 ValgrAI - Valencian Graduate School and Research Network of Artificial Intelligence, Valencia, Spain; Lodz University of Technology: Politechnika Lodzka, POLAND

## Abstract

In this study, we introduce an innovative methodology for anomaly detection of curves, applicable to both multivariate and multi-argument functions. This approach distinguishes itself from prior methods by its capability to identify outliers within clustered functional data sets. We achieve this by extending the recent AA + *k*NN technique, originally designed for multivariate analysis, to functional data contexts. Our method demonstrates superior performance through a comprehensive comparative analysis against twelve state-of-the-art techniques, encompassing simulated scenarios with either a single functional cluster or multiple clusters. Additionally, we substantiate the effectiveness of our approach through its application in three distinct computer vision tasks and a signal processing problem. To facilitate transparency and replication of our results, we provide access to both the code and the datasets used in this research.

## 1 Introduction

In functional data analysis (FDA), each observation is a function. These functions can be univariate or multivariate, with one or more arguments. A classic reference in this field is [1]. In FDA, as in multivariate analysis (MVA), anomaly detection is an essential step [2]. However, handling outliers in functional data is challenging due to its infinite-dimensional nature [3]. [4] proposed a taxonomy of functional outliers, which was improved by [5]. According to this taxonomy, outliers can be isolated or persistent depending on whether the anomalous behavior occurs during a very short or a long part of the function domain, respectively. Furthermore, persistent outliers could be categorized into shift, amplitude or shape outliers depending on whether they are identical to the other observations after a baseline correction, a rescaling or a warping transformation, respectively.

Nevertheless, this taxonomy does not consider the case of functional clustered data. Clustered data are characterized as data that can be classified into a number of distinct groups or clusters. When we work with clustered data, an upper level in the hierarchy of that taxonomy is the “degree of zoom with which we view the data”. In that case, functional anomalies can be classified as global or local anomalies. A global functional outlier is an anomalous function with respect to all other observations in the data set as a whole, although not necessary with respect to their neighboring observations. On the contrary, a local functional outlier is an anomalous function with respect to other neighboring observations, although it may not be an anomaly in a global view of the data set. By neighboring observations to a certain function *f*, we mean other functions in the data set within a certain distance from *f*. This distance can be the *L*^2^ distance between two functions. Therefore, it is always assumed that our functions belong to a Hilbert space, satisfy reasonable smoothness conditions and are square integrable functions on their domain, a certain interval [*a*, *b*].

**Fig 1:**
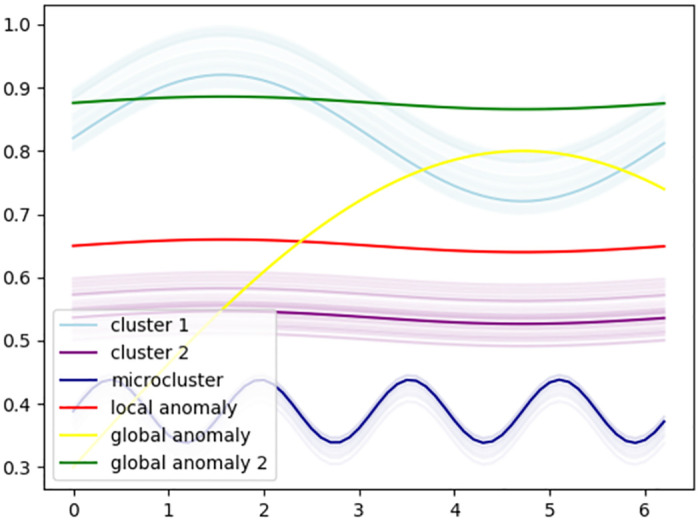
Example illustrating clustered functional data with two global anomalies, a local anomaly and a micro-cluster.

Fig 1 illustrates the ideas presented in the previous paragraph, which is a generalization of the concept of global and local anomalies in MVA [6]. There are two clear clusters (*c*1 and *c*2 in light blue and purple, respectively) and outliers (*f*1 in yellow and *f*2 in green). Clusters appear with transparent colors, except one random function of each cluster that is opaque. *f*1 and *f*2 are global anomalies. *f*1 is very different from the other data (its distance to the other functions is very large), as is *f*2, which can be seen as a shape outlier for *c*1 or a vertical shift outlier for *c*2. However, *f*3 (shown in red) is a local anomaly. When focusing on the data set globally, *f*3 can be considered a normal case because it is not too far from cluster *c*2. But when we compare *f*3 with its neighbors, i.e., the functions in cluster *c*2 while neglecting all the other observations, *f*3 can be seen as an anomalous record. Depending on the applications, local anomalies may or may not be of interest. Another situation appears in Fig 1. The functions in *c*3 in dark blue generate an open question: Are the observations in *c*3 anomalies or a (small) regular cluster? *c*3 constitutes what is called a micro-cluster. For these cases, it can be very useful that our anomaly detection algorithm returns outlierness scores, so that the degree of outlierness of the functions in *c*3 should be larger than for normal records, but smaller than for clear outliers.

As regards a taxonomy about unsupervised anomaly detection algorithms for MVA, [6] divided them into four categories: (1) Nearest-neighbor (NN) based techniques, (2) Clustering-based methods, (3) Statistical algorithms and (4) Subspace techniques. Despite the great variety of methods in MVA for outlier detection, in the more recent field of FDA, many functional outlier detection methods are global depth-based [7]. Therefore, there are many possibilities to extend methods that perform well in MVA to FDA for outlier detection.

In the sphere of multivariate analysis (MVA), while depth-based methods offer valuable insights, they may exhibit limitations, particularly in detecting anomalies within the sparse interior regions of a data set, as noted by [2]. These methods, in their conventional form, often face challenges when tasked with identifying outliers within clustered data. This characteristic suggests that their efficacy may be compromised when applied to functional data exhibiting similar clustering traits. To our knowledge, the specific issue of outlier detection in clustered data, a notable concern in MVA, has not been extensively examined within the functional data analysis (FDA) framework. Addressing this gap forms one of the primary objectives of our present study. It is is well known that unsatisfactory results can be obtained when clustering methods that are no robust are applied in the presence of outliers [8]. For example, one group could be consititued by outlying observations or heterogeneous groups could be joined together [9]. Therefore, the naive approach of using no robust clustering methods and looking for outliers inside the groups is discarded.

To achieve this primary objective, another aim is to extend a recent technique that performs well for detecting outliers in MVA to FDA for univariate and multivariate functions, with one or more arguments, and compare it with previous alternatives. This MVA technique was proposed by [10] and performed among the best in an extensive comparison with 23 state-of-the-art outlier detection algorithms in MVA with several benchmark data sets. The technique combines categories (1) and (4), since it projects data into relevant subspaces by archetype analysis (AA) [11] and then uses an NN-technique through an appropriate ensemble of the results. Despite NN-based techniques being very popular in MVA for outlier detection, they have not been very widely used in FDA [7]. In this work, we propose to use functional archetypal analysis (FAA) [12], since projecting in appropriate subspaces can improve proximity-based methods, and then to use NN-techniques to detect outliers in those subspaces. AA represents the instances by means of a mixture of archetypes, which are a mixture of instances. The objective of FAA is analogous, but with functions. AA is sensitive to outliers since archetypes lie on the boundary of the convex hull of the data set. The idea of the projections into AA (or FAA) is to exploit this sensitivity to outliers for detecting them. Although it is not the first time that AA or archetypoid analysis (ADA), which is a variant of AA [13], have been used for outlier detection of functions [7, 14], in previous cases, they assumed that the sample is constituted by homogeneous functions generated from a unimodal distribution, i.e., these techniques will fail for clustered data.

To highlight the challenge of finding outlier of functional data (FD) we must bear in mind that FD are not only high-dimensional data, but intrinsically infinite dimensional [15]. Many of the popular anomaly detectors do not work properly with increasing dimensionality. This is an artifact of the well-known curse of dimensionality, whose impact in the outlier detection problem was first noted in [16]. For example, the effectiveness of proximity-based detection in increasing dimensionality is compromised because distances in the original space lose descriptive power. Therefore, a mapping to some more suitable space is necessary, which is called projected outlier detection or, alternatively, subspace outlier detection [2]. There are different classes of projections, such as rarity-based, unbiased and aggreagtion based methods [2, Ch. 5]. The main novelty of this work lies in the use, for the first time, of FAA as a rarity-based subspace method for functional outlier detection. Rarity-based methods seeks subspaces based on rarity of the underlying distribution. Anomaly detection process can be enhanced by identifying subspaces in which the observations deviate remarkably from the normal behavior. Note that the objective of FAA is to discover *p* extreme functions and multiple diverse subspaces can be obtained by changing FAA parameter *p*, i.e. by parametric ensemble. Therefore, FAA subspace outlier detection is inherently posed as an ensemble-centric problem. After each different FAA projection, we consider a proximity-based methodology and a model-centric ensemble, but these base detectors could be replaced by others. Previous methods [7, 14] did not use FAA as parametric ensemble, but they only considered FAA with a unique parameter, *p* = 3, and did not exploit the information in this projection as in our proposal.

This study makes several significant contributions to the field of functional data analysis. We present an innovative method for outlier detection that is applicable to both univariate and multivariate functions with varying numbers of arguments, a notable advancement over many existing methods that are limited to univariate functions. Unlike other methods, our proposal does not require to fix the proportion of outlier detection. Our method is thoroughly evaluated through a simulation study, where it is compared against a comprehensive suite of the most advanced techniques in functional outlier detection. This rigorous comparison reveals the enhanced performance of our approach, particularly in scenarios involving functional clustered data—a situation in which previous methods have shown limitations and it is really challenging [17].

Furthermore, we extend the practical application of our proposed method to real-world data, exemplifying its efficacy in resolving complex problems within the realm of computer vision and signal processing. The application to these real-world problems not only demonstrates the versatility of our method but also its superiority in practical settings.

In line with our commitment to the scientific community’s principles of transparency and reproducibility, we are releasing the code that underpins our methodology. This will allow peers and future researchers to replicate our findings and extend upon our work, reinforcing the integrity and applicability of our contributions.

The paper is organized as follows. Section 2 reviews AA, FAA and previous functional outlier detection methods together with robust curve clustering methods. Section 3 introduces our proposal. The simulation study and results using real data are presented and discussed in Section 4. Finally, we provide some conclusions in Section 5.

## 2 Background

### 2.1 AA and FAA

In MVA, let **X** be an *n* × *m* data matrix with *n* observations (*x*_*i*_) and *m* variables. The rows of a *p* × *m* matrix **Z** contain the *p* archetypes (*z*_*j*_), which are a mixture of data points, while data points are in turn approximated by a mixture of archetypes. Therefore, we have to minimize the following residual sum of squares (RSS), where the elements of the two *n* × *p* matrices *α* and *β* have to be determined:

$$\begin{array}{c} \begin{array}{c} RSS=\sum_{i=1}^{n}{\left\|x_{i}-\sum_{j=1}^{p}\alpha_{ij}z_{j}\right\|}^{2}=\\ \sum_{i=1}^{n}{\left\|x_{i}-\sum_{j=1}^{p}\alpha_{ij}\sum_{l=1}^{n}\beta_{jl}x_{l}\right\|}^{2}, \end{array} \end{array}$$ (1)

with two constraints: 1) $\sum_{j=1}^{p}\alpha_{ij}=1$ with *α*_*ij*_ ≥ 0 and *i* = 1, …, *n*, and 2) $\sum_{l=1}^{n}\beta_{jl}=1$ with *β*_*jl*_ ≥ 0 and *j* = 1, …, *p*. In summary, *α*_*ij*_ is the weight of the archetype *z*_*j*_ in the approximation of *x*_*i*_, ${\hat{x}}_{i}=\sum_{j=1}^{k}\alpha_{ij}z_{j}$; while *β*_*jl*_ is the weight of the data point *x*_*l*_ in the definition of the archetype $z_{j}=\sum_{l=1}^{n}\beta_{jl}x_{l}$. AA can be computed by an alternating minimizing algorithm described by [11]. This is a nondeterministic algorithm, therefore it is run beginning from 10 random initializations, and the best model is chosen for each *p*.

In FDA, let {*x*_1_(*t*), …, *x*_*n*_(*t*)} with *t* ∈ [*a*, *b*] be the FD set. In FAA, RSS is computed with a functional norm (the *L*^2^-norm, ${\left\|x\right\|}^{2}=<x,x>=\int_{a}^{b}x{\left(t\right)}^{2}dt$), since the observations (*x*_*i*_(*t*)) and archetypes (*z*_*j*_(*t*)) are now functions. However, the matrices *α* and *β* are interpreted as in AA. As functions are recorded at discrete sites in practice, we can apply AA to the function values of *m* equally-spaced values from *a* to *b* to calculate FAA. For a more computationally efficient approach, FD can be approximated by means of basis functions: $x_{i}(t)\approx \sum_{h=1}^{m}b_{i}^{h}B_{h}\left(t\right)$, where *B*_*h*_ (*h* = 1, …, *m*) are the basis functions and **b**_*i*_ is the vector of length *m* with the coefficients. So, the RSS in FAA is [12]:

$$\begin{array}{c} \begin{array}{c} RSS=\sum_{i=1}^{n}{\left\|x_{i}-\sum_{j=1}^{p}\alpha_{ij}z_{j}\right\|}^{2}=\\ \sum_{i=1}^{n}{\left\|x_{i}-\sum_{j=1}^{p}\alpha_{ij}\sum_{l=1}^{n}\beta_{jl}x_{l}\right\|}^{2}=\sum_{i=1}^{n}{\text{a}}_{i}^{\prime }\text{W}{\text{a}}_{i}, \end{array} \end{array}$$ (2)

where ${\text{a}}_{i}^{\prime }=b_{i}^{\prime }-\sum_{j=1}^{p}\alpha_{ij}\sum_{l=1}^{n}\beta_{jl}b_{l}^{\prime }$ and **W** is the order *m* symmetric matrix with the inner products of the pairs of basis functions $w_{m_{1},m_{2}}=\int B_{m_{1}}B_{m_{2}}$. **W** is the order *m* identity matrix when the basis is orthonormal, such as the Fourier basis. In that case, we can obtain FAA by using AA with the basis coefficients. In another case, we have to compute **W** one time only by numerical integration.

If our data set is composed of multivariate functions, in particular to simplify the explanation, bivariate functions **f**_*i*_(*t*) = (*x*_*i*_(*t*), *y*_*i*_(*t*)), with coefficient vectors for the basis functions *B*_*h*_, ${\text{b}}_{i}^{x}$ and ${\text{b}}_{i}^{y}$, respectively; then the RSS is as follows:

$$\begin{array}{c} RSS=\sum_{i=1}^{n}\left|\right|{\text{f}}_{i}-\sum_{j=1}^{p}\alpha_{ij}{\text{z}}_{j}{\left|\right|}^{2}=\\ \sum_{i=1}^{n}\left|\right|{\text{f}}_{i}-\sum_{j=1}^{p}\alpha_{ij}\sum_{l=1}^{n}\beta_{jl}{\text{f}}_{l}{\left|\right|}^{2}=\\ \sum_{i=1}^{n}\left|\right|x_{i}-\sum_{j=1}^{p}\alpha_{ij}\sum_{l=1}^{n}\beta_{jl}x_{l}{\left|\right|}^{2}\\ +\sum_{i=1}^{n}\left|\right|y_{i}-\sum_{j=1}^{p}\alpha_{ij}\sum_{l=1}^{n}\beta_{jl}y_{l}{\left|\right|}^{2}=\\ \sum_{i=1}^{n}{{\text{a}}^{x}}_{i}^{\prime }{\text{Wa}}_{i}^{x}+\sum_{i=1}^{n}{{\text{a}}^{y}}_{i}^{\prime }{\text{Wa}}_{i}^{y} \end{array}$$ (3)

where ${{\text{a}}^{x}}_{i}^{\prime }={{\text{b}}^{x}}_{i}^{\prime }-\sum_{j=1}^{p}\alpha_{ij}\sum_{l=1}^{n}\beta_{jl}{{\text{b}}^{x}}_{l}^{\prime }$ and ${{\text{a}}^{y}}_{i}^{\prime }={{\text{b}}^{y}}_{i}^{\prime }-\sum_{j=1}^{p}\alpha_{ij}\sum_{l=1}^{n}\beta_{jl}{{\text{b}}^{\text{y}}}_{l}^{\prime }$. As before, for orthogonal basis functions, FAA can be obtained by applying AA to the *n* × 2*m* coefficient matrix composed by joining the coefficient matrix for *x* and *y* components.

### 2.2 Functional outlier detection methods

We review some of the main methods devoted to functional outlier detection along with their implementation in R [18]. These methods will be compared with our proposal. [19] used a likelihood ratio test (LRT). [20] identified outliers as functions whose depth levels are below a certain threshold. This threshold is established by a bootstrap procedure based on either trimming (TRIM) or weighting of the sample (POND). LRT, TRIM and POND are found in the R packages **fda.usc** [21] and **rainbow** [22]. In this package, we also find the procedure by [23] (ISFE), where integrated square forecast errors are used; the procedure by [24] (RMAH), where the robust Mahalanobis distance is applied but regarding the functions as multivariate observations; and the functional highest density region (HDR) boxplot introduced by [25]. The classical boxplot was extended to FD by [26]. This procedure (FB) is available in the R package **fda** [27]. The outliergram (OUG) was proposed by [28] and can be found in the R package **roahd** [29]. [30] proposed to use a kernelized functional spatial local depth for identifying outliers. The Functional Outlier Map (FOM) was proposed by [4] and improved by [31]. It is based on functional outlyingness measures and is available in the R package **mrfDepth** [32].

More recent literature comprise methods by [33] based on functional directional outlyingness; [34] based on functional principal component analysis and an adaptive and data driven approach for dimension selection; [35], where a sequence of transformations was considered; and [36], where the total variation depth was proposed. [37] considered features extracted from differential geometry. Outlier detection for multivariate time series is carried out by [38] using a functional approach, through quantile cross-spectral densities and functional depths. Other methods are based on elastic depths [39] and a new elastic metric [40]. Methodologies for detecting outliers in big functional data have recently been proposed by [7, 41–43]. This last method, CRO-FADALARA, is explained below. Depthgram was introduced by [44] for visualizing outliers in high-dimensional functional data. Depending on the problem, other works aim to develop robust methods against outliers, such as [3].

Two previous methods, FOADA and CRO-FADALARA, used ADA for functional outlier detection, although in different ways to our proposal. On the one hand, FOADA was introduced by [14]. In FOADA, outliers were iteratively detected by computing ADA with three archetypoids repeatedly while sieving the sample. The outlier detection is based on the alpha coefficients either using the robust Mahalanobis distance (FOADARMAH) or using a threshold for the alpha values (FOADATH). FOADA iterates until no more outliers are detected. On the other hand, CRO-FADALARA has two phases: in the first phase, the cleaning phase, the most obvious outliers (amplitude and isolated) are detected with the classical boxplot, while in the second phase, robust ADA is computed and outliers are detected by applying the adjusted boxplot [45] to the norm of the residuals. Note the differences with our proposal, which is not an iterative procedure like FOADA nor does it use the residuals like CRO-FADALARA. As explained in Sec. 1, our proposal is a rarity-based subspace outlier detection method, a parametric-ensemble method.

As we deal with clustered functional data sets, we also consider in the comparison a classical reference for robust clustering such as [46], TRIMKMEANS, which can be found in the R package **trimcluster** [47]. Trimming techniques have been used in functional data contexts, such as the works by [8, 48–50]. Proportion of observations to be trimmed have to specified. Trimmed observations will be considered as outliers. [51] performed hierarchical functional clustering with special consideration of outliers and [17] considered a contaminated mixture model.

Some interesting applications of functional outlier detection are flood analysis [52], monitoring of helicopters in flight and the analysis of the spectrometry of construction materials [53], traffic flow data [54], bike sharing system data [55] and profile monitoring in industrial manufacturing [56].

## 3 The FAA + *k*-NN method for detecting functional outliers

As mentioned in Section 1, the objective is to project FD in relevant subspaces, where outliers can be better detected, and to reduce the dimensionality. Then distance-based methods are applied to detect anomalies in those subspaces. FAA is considered for projection, with different *p* values (i.e. number of archetypes), since archetypes do not necessarily nest nor are they orthogonal to one another [11]. Therefore, for each *p*, the returned archetypes can be changed to better capture the configuration of the data set. This property, together with the fact that the archetypes are on the boundary of the convex hull of the data for more than one archetype [11], favors our proposal. On the one hand, as we can obtain different explanations of the data with different number of archetypes, we can combine the different results by ensembles. This is advantageous since accuracy and diversity are desirable properties for the good performance of ensembles [57]. On the other hand, as the number of archetypes increases, some returned archetypes could be outliers, but this is not a problem for us; quite the opposite, in fact. Once the FD set is FAA projected for multiple number of archetypes, the *k*-NN method is applied to the *α* values for each projection (remember that the *α* values always add 1, regardless of the number of archetypes).

In conclusion, our proposed methodology is encapsulated within two primary steps:

**Step 1** Project FD in relevant subspaces using FAA.
  1. Project the functional data (FD) into the relevant subspaces by applying FAA. Begin with a single archetype (*p* = 1) and incrementally increase the number of archetypes up to the maximum number predetermined for your study (*p* = *p*_2_).
  2. Calculate the RSS for each value of *p* as you progress from *p* = 1 to *p* = *p*_2_.
  3. Construct a plot of RSS against the corresponding values of *p*.
  4. Examine the plot to identify the ‘elbow’, which is the point where the RSS begins to decrease at a diminishing rate as *p* increases. This is achieved using the elbow criterion, an intuitive heuristic approach used elsewhere [11].
  5. Select the number of archetypes corresponding to the ‘elbow’ as the minimal number of archetypes (*p*_1_) that provides a satisfactory description of the data. This represents the point at which additional archetypes yield only marginal improvements in data representation.
**Step 2** Apply *k*-NN (sum of Euclidean distance to *k* nearest neighbors) to detect anomalies.
  1. Apply the *k*-NN algorithm to each of the *α* matrices for the subspaces defined by *p*_1_ up to *p*_2_. Use the sum of Euclidean distances to the *k* nearest neighbors to calculate the outlier scores for each data point.
  2. Normalize the outliers scores of each data point by implementing a cumulative-sum approach, which is equivalent to averaging the outlier scores across the different projections. This results in a single outlier score for each data point that synthesizes the information from all the projections.
  3. As the same *k* is used each time, the scale of the outlier scores is the same, so the aggregation ensemble is well founded.

Note that if dealing with multivariate FD with different ranges in each functional variable, a previous standardization of the data may be necessary.

As the bias-variance trade-off in outlier detection is almost identical to that in classification [2], averaging also reduces variance in outlier identification. If we want to make the algorithm more robust, we could apply the Step 2 for a range of *k* and then averaging the results.

Bias-variance theory developed in the supervised learning field can also be adjusted to anomaly detection with some changes [58]. [2, Ch. 6] details the similarities between outlier ensembles and classification ensembles. Our proposal consists of a rarity-based subspace outlier detection method. It is a parametric ensemble, where a range of different values of *p* are used with FAA in step 1, and then the scores obtained in step 2 are averaged. This is a typical outlier ensemble approach [59]. Outlier ensembles are especially convenient in settings in which they are able to reduce the uncertainty related with difficult algorithmic selections [2, Ch. 6], such as discovering relevant subspaces to different observations. This makes FFA very suitable for this purpose since FAA results for different *p* values are not necessarily nested and they change to explore better the shape of the dataset. Furthermore, averaging the scores in step 2 results in variance reduction, which improves performance by the theoretical foundations explained by [58] for outlier ensembles. For the same reason, different *k* values can be considered in step 2, and their results can be averaged, as explained before.

By default, we obtain outlier scores, where the highest level of outlierness is indicated by the highest scores. If we would like to return a binary label (being an outlier or not), a box-plot with the outlier scores could be used and we could label the points identified as outliers as anomalies. Despite being a simple hardening strategy, it has been proven effective in our experiments.

This is an extension of the proposal by [10] from the multivariate case to the functional case, i.e., in Step 1, we use FAA instead of AA. As aforementioned, [10], performed among the best in an extensive comparison with 23 state-of-the-art outlier detection algorithms in MVA with several benchmark data sets. One of those outlier detection algorithms was using only the *k*-NN method, i.e. skipping step 1. Another one was substituting the step 1 by robust principal component analysis (RPCA) and using PC scores with the *k*-NN method. Using AA as step 1 improved clearly the detection of anomalies than skipping step 1 or using RPCA.

### 3.1 Interpretation of parameters

The parameters within our proposed framework are pivotal to the analysis and are interpreted as follows:

- *p*_1_: This parameter signifies the ideal number of archetypes that adequately describe the dataset, i.e the point where the elbow is found.
- *p*_2_: Set to be larger than *p*_1_, *p*_2_ defines the upper limit of the number of archetypes to consider. The choice of *p*_2_ should be reflective of meaningful improvements in model accuracy. Increasing *p*_2_ without concurrent improvements in loss reduction does not add value to the analysis and may unnecessarily complicate the model.
- *k*: Determines the sensitivity to the size of clusters identified as outliers. A cluster exceeding the size of *k* will not be flagged by the algorithm using the *k*-NN method, since *k* points will be inherently proximal. It is recommended to employ a range of *k* values, as suggested in previous works [6].

#### 3.1.1 Toy example

**Fig 2:**
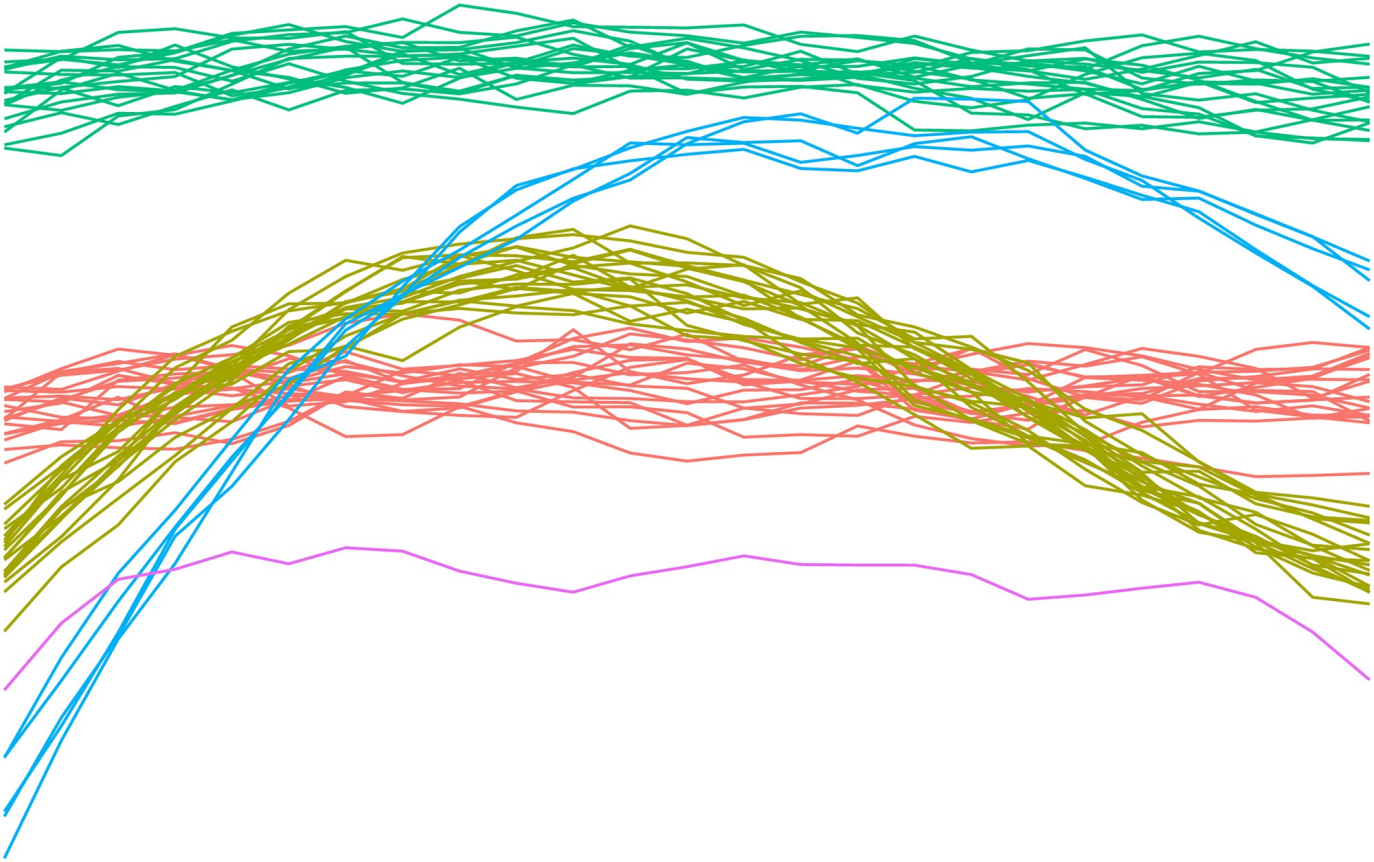
Example of functional data set containing multiple clusters.

To elucidate the parameter interpretation within our framework, we introduce an illustrative example featuring five distinct distribution types. Fig 2 illustrates that the dataset comprises three larger clusters (with 20 elements each), a smaller cluster (containing 5 elements), and a lonely outlier.

**Fig 3:**
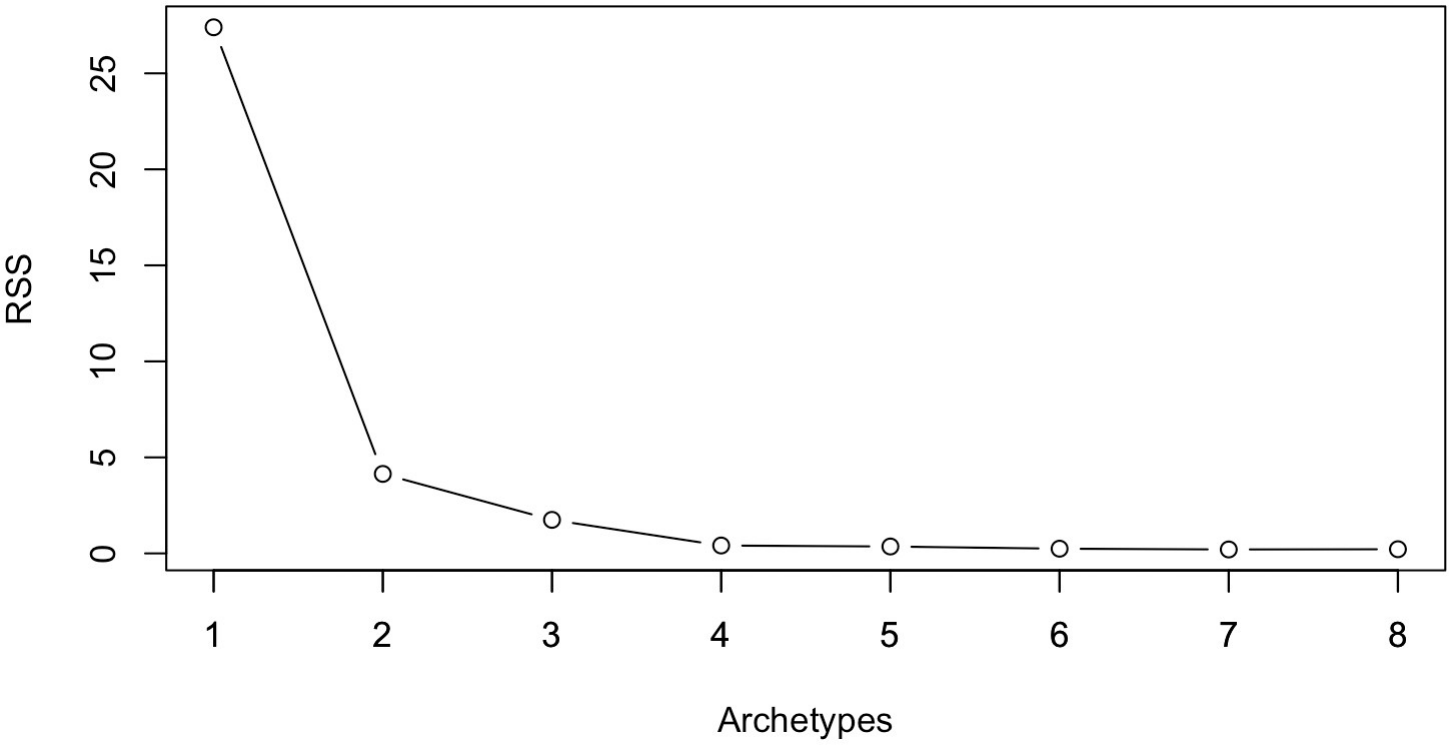
The Residual Sum of Squares (RSS) plotted against the number of archetypes for the toy example.

In alignment with our methodology, the initial task is to compute data projections through FAA. In this example, we have chosen *p*_max_ = 8, a decision justified in Fig 3, which validates its sufficiency for the determination of *p*_1_ and *p*_2_.

Upon calculating the projections, Fig 3 shows the RSS plotted against the number of archetypes. The ‘elbow’, indicative of the optimal archetype count, is apparent at two archetypes, thereby setting *p*_1_ = 2. Additionally, the plot indicates a minimal RSS variance beyond four archetypes, leading to the selection of *p*_2_ = 4.

**Fig 4:**
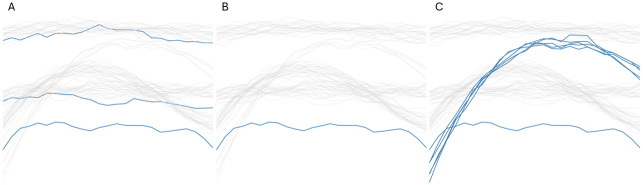
Detection of outliers using different values of *k*. Outliers appear in blue. A: *k* = 1. B: 1 ≤ *k* ≤ 5. C: 5 < *k* ≤ 10.

With the projection range (*p*_1_ ≤ *p* ≤ *p*_2_) determined, we advance to identifying an appropriate *k* value that will facilitate the detection of desired outliers. In Fig 4, we illustrate three configurations of *k*, each yielding distinct outcomes.

In the initial scenario presented in Fig 4A, the parameter *k* is set to 1. This choice is based on the intention to identify outliers that are singular, i.e., data points that stand alone. Contrary to the expectation of detecting only the solitary purple data point as an outlier, the analysis reveals a total of three outliers. This outcome suggests the presence of additional lonely outliers within the data set.

Moving to the second scenario, depicted in Fig 4B, the *k* parameter is varied from 1 to 5. This range is strategically chosen to enhance the robustness of the outlier detection process, aiming to mitigate the impact of local anomalies that might otherwise be mistaken for significant outliers. In this case, only the purple data point is considered as outlier, indicating that the other two could be local anomalies.

In the concluding scenario, shown in Fig 4C, the strategy diverges from isolating individual outliers to recognizing clusters of outliers consisting of up to five data points by opting for a *k* value between 5 and 10. Therefore, the methodology also categorizes the cluster of five data points as an outlier.

In conclusion, the selection of the parameters *p*_1_ and *p*_2_ is informed by the data illustrated in Fig 3. In addition, the choice of the *k* value hinges on the specific outlier definition one wishes to apply and the particular characteristics of outliers that need to be identified. This exposition has clarified the implications of parameter choices and illustrated their utility in configuring outlier detection for diverse situational requirements.

## 4 Results and discussion

### 4.1 Simulation study

**Fig 5:**
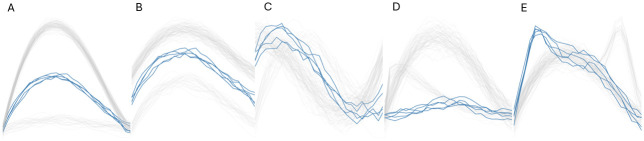
Scenario 2: Clustered data samples with different types of outliers shown in blue. A: Amplitude. B: Vertical shift. C: Horizontal shift. D: Shape. E: Isolated.

Our method is assessed in two different scenarios, considering non-clustered or clustered data. In each scenario, five kinds of outliers contaminate the models: amplitude, vertical shift, horizontal shift, shape and isolated outliers. In all of the following models, *ϵ*(*t*) will be a Gaussian process with zero mean and covariance function *γ*(*s*, *t*) = 0.3 exp{−|*s* − *t*|/0.3}, and functions are observed at 25 equidistant points between 0 and 1. In the first scenario, there is a single cluster and the simulation design was similar to that used by [7, 14, 20, 28, 60]. Cluster 1 is defined by the equation *X*_1_(*t*) = 30*t*(1 − *t*)^3/2^ + *ϵ*(*t*). In the second scenario, a second cluster is added. We generate samples from all models with a sample size equal to 100. In the first and second scenario, the number of outliers is 5, while the number of functions in the first cluster is 70 (95) in the second (first) scenario and 25 functions make up the second cluster, respectively. The simulation design of outliers and cluster 2 depends on the kind of outliers assessed and appears in Table 1. Fig 5 displays the simulated functions for scenario 2.

**Table 1:** Simulation design of outliers for scenarios 1 and 2 (first row) and cluster 2 in scenario 2 (last row). *Z*_1_ denotes a truncated standard normal density, centered in the 6th observation, while *Z*_2_ denotes a truncated standard normal density, centered in the 20th observation, i.e., they are defined for the first 11 observed points and the last 11 observed points, respectively.

	Amplitude	V-shift	H-shift	Shape	Isolated
Outliers	3*X*_1_(*t*)	3 + *X*_1_(*t*)	*X*_1_(*t* + 0.15)	15*t*^2^(1 − *t*)^2^ + *ϵ*(*t*)	*X*_1_(*t*) + 15*Z*_1_
Cluster 1	*X*_1_(*t*)	*X*_1_(*t*)	*X*_1_(*t*)	*X*_1_(*t*)	*X*_1_(*t*)
Cluster 2	6*X*_1_(*t*)	6 + *X*_1_(*t*)	*X*_1_(*t* + 0.3)	10*t*^1/3^(1 − *t*)^3^ + *ϵ*(*t*)	*X*_1_(*t*) + 15*Z*_2_

We have generated 50 synthetic data sets and applied FAA + *k* − NN together with the methods explained in Section 2.2. Default parameters (they can be seen in the code file in the Supplementary material) have been considered, except for the procedures that require the outlier rate to be specified. For those procedures (LRT, TRIM, POND and HDR), the true values have been used, which could give them an advantage. For TRIMKMEANS one and two clusters are considered for the first and second scenario, respectively. In accordance with our methodological procedure, we have established for our method that *p*_1_ = 2, *p*_2_ = 5, and *k* ranges from 5 to 15. This last parameter is based on our knowledge that the outliers will consist of a group of 5 elements.

**Fig 6:**
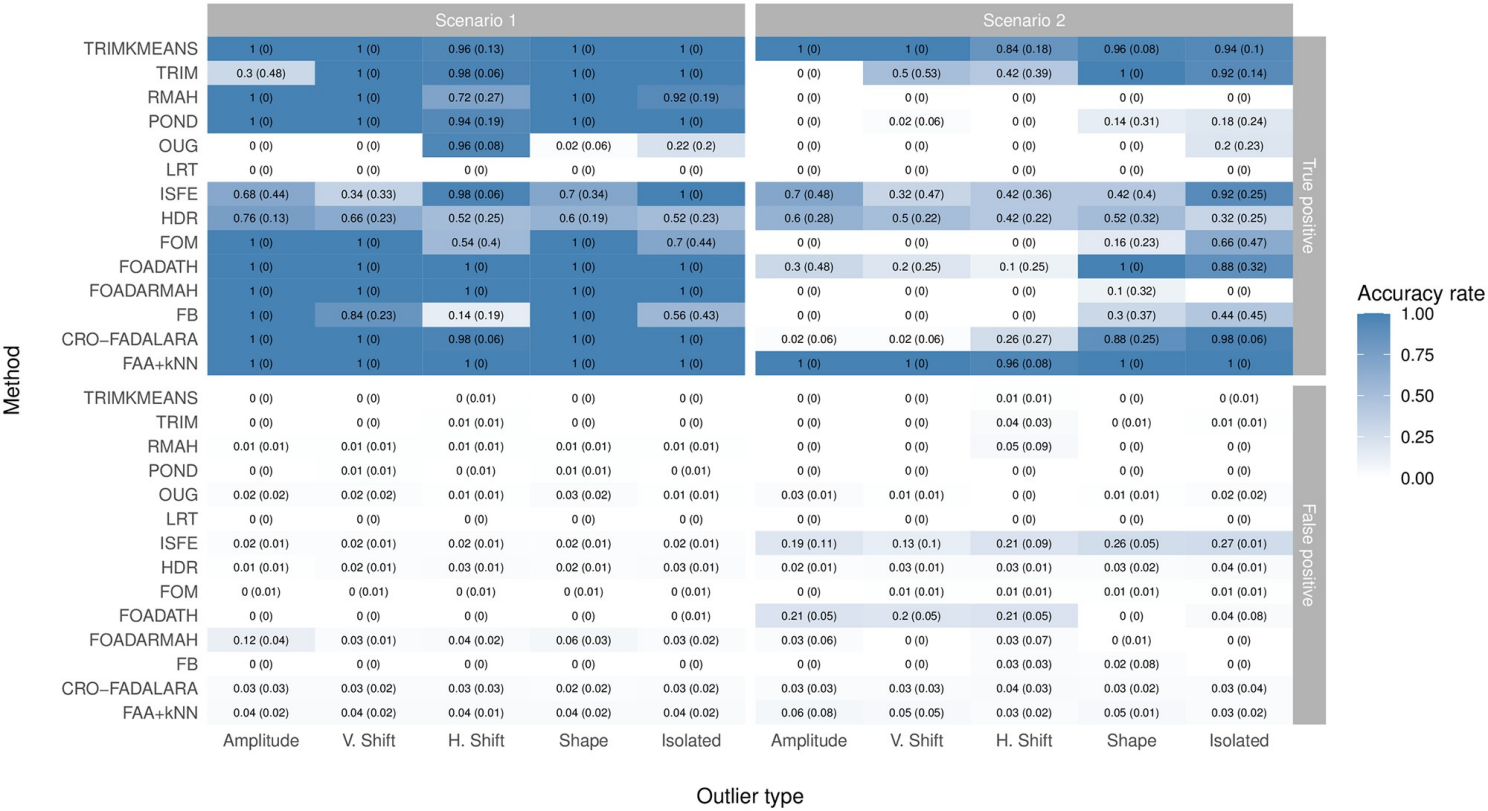
Mean and standard deviation (in brackets) of the proportion of correctly (True positive) and falsely (False positive) identified outliers over 50 simulation runs for Scenario 1 and 2.

The results are summarized in Fig 6. In scenario 1, our proposal obtains 100% correct detection of outliers for all the types, with 4% false positive detection. These results are only improved by FOADATH, which gives no false negatives. FOADARMAH also detects all the outliers of all types, but with a slight increase in false negatives for some types. For the other methods, perfect detection is obtained for some types, but not for others, such as CRO-FADALARA, TRIMKMEANS, RMAH, ISFE, TRIM, POND, FOM and FB, while for other methods, the percentage of correct detection is lower in all the classes, such as OUG and HDR. As regards the results in scenario 2, our proposal is clearly the best. FAA+*k*NN detects all the outliers for all types of the outliers, except for H-shift, with 96% success and a very low false positive rate. The accuracy of TRIMKMEANS is very high, but it is smaller than that of our method for H-shift, shape and isolated outliers, whose accuracies are 84%, 96% and 94%, respectively. The other methods cannot detect any outlier in many cases. The third-best method for amplitude outliers is ISFE, with 70% correct detection but 19% false positives. The third-best methods for V-shift outliers are TRIM and HDR, with 50% correct detection. The third-best methods for H-shift are ISFE, TRIM and HDR, with 42% correct detection. For shape outliers, FOADATH and TRIM also obtain 100% correct detection. For isolated outliers, the best-second method is CRO-FADALARA, with 98% correct detection.

### 4.2 Real data

In addition to simulations, we tested our proposed method on three datasets from image data and another from signal data. The first two sets of data include univariate curves, the third one involves three-variate functions and the fourth one includes three-variate functions with two arguments.

Since this study involves analysis of existing and publicly data sources coming from peer-reviewed and formally published sources, which are detailed in the following sections, consent was waived for this study for data with human participants. Furthermore, the statistical analyses carried out in this study are completely different from those sources and any other, since those analyses are made with our original methodology proposed in Section 3. Therefore, this study does not constitute dual publication.

#### 4.2.1 Planes dataset

The shape database for aircraft silhouette recognition curated by [61] encompasses a diverse array of fighter aircraft, including the Mirage, Eurofighter, F-14, Harrier, F-22, and F-15. Notably, the F-14 Tomcat is represented by two distinct configurations due to its variable-sweep wing design, which can be either extended or retracted, thereby contributing to the overall count of seven shape classes within the database.

The database was meticulously compiled by photographing scale models of these aircraft from a top-down perspective, resulting in images with a resolution of 640x480 pixels. These images were subsequently processed using the Spedge and Medge techniques for color image segmentation, facilitating the isolation of the aircraft shapes from the background.

Despite the precision in capturing and processing the images, the resultant shapes were not immune to imperfections. Artifacts arising from the segmentation process introduced noise, while variations in the viewing angles during photography led to distortions in the perceived shape of the aircraft. To refine the database, the shapes underwent additional processing with a Gaussian filter, characterized by a standard deviation of 10, to smooth out irregularities. Furthermore, a standardization step was employed, normalizing the length of the shape contours to 144 points to maintain consistency across the dataset.

The dataset encapsulates a total of 105 samples, with the distribution across the seven classes being 15, 14, 9, 16, 13, 18, and 20, respectively.

**Fig 7:**
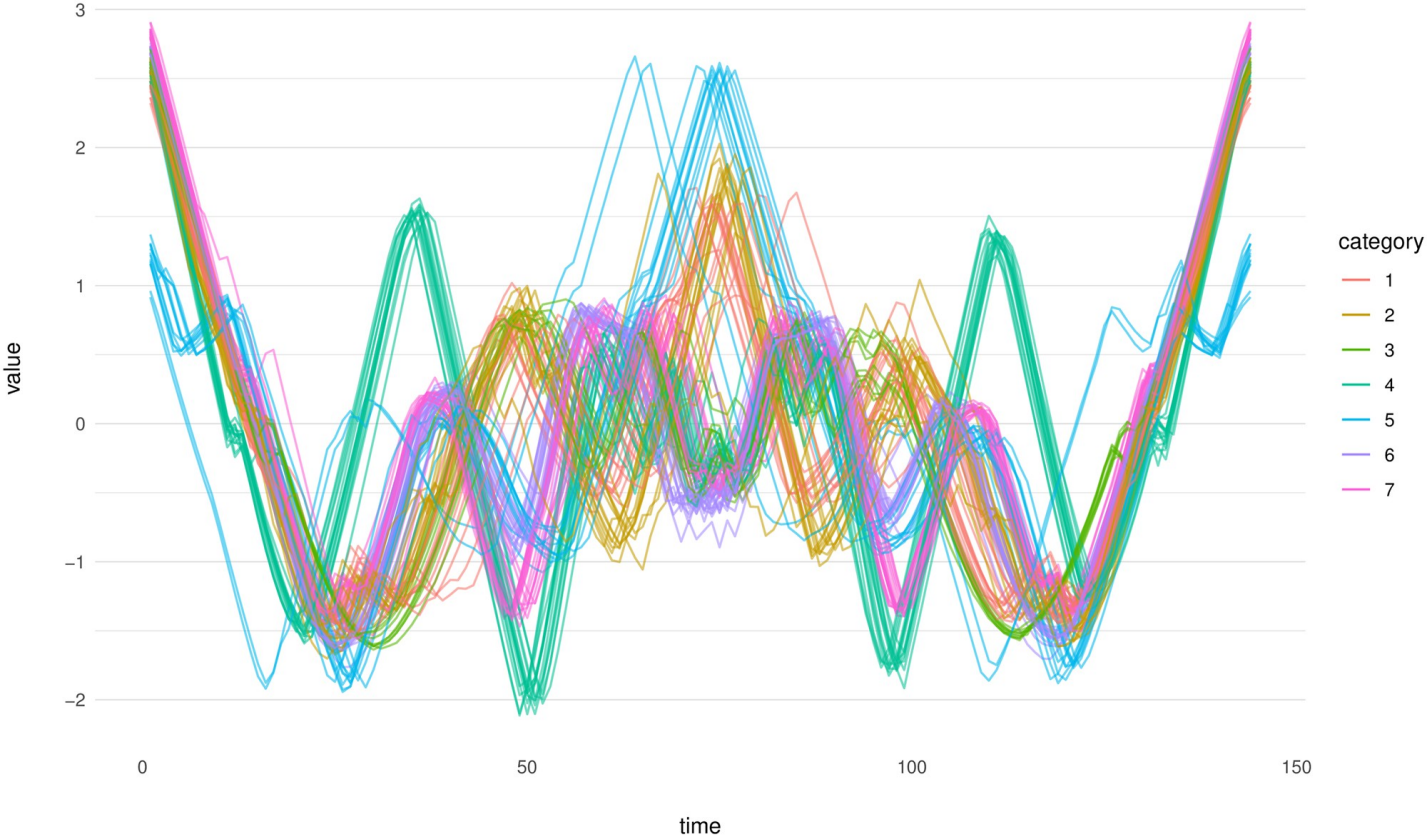
Planes data set. Planes belonging to each of the seven classes are represented in a different color according to the legend.

Fig 7 provides a visual depiction of the shapes of the various fighter aircraft within the database. At first glance, we could see that this dataset includes some anomalies; for instance, in class 5, certain shapes are shift outliers.

**Fig 8:**
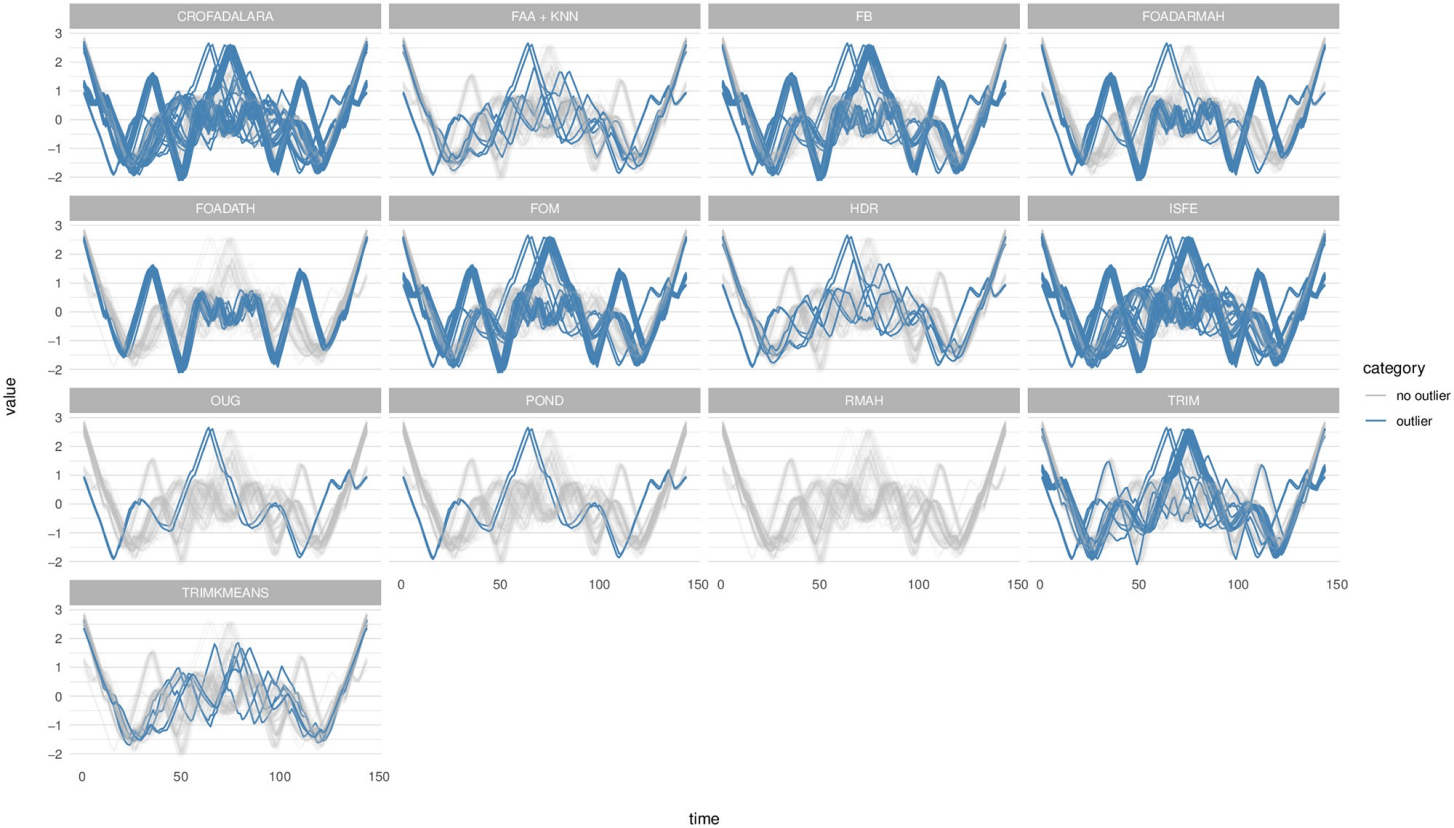
Outliers detected in the planes data set for the different methods.

We sought to pinpoint outliers by applying previously outlined methods, factoring in an outlier rate of 5% for those who require it, and utilizing their default settings. For TRIMKMEANS, 7 groups are considered. In our approach, we refer to the RSS plot to select values for *p*_1_ and *p*_2_. Accordingly, *p*_1_ is set to 7 and *p*_2_ to 11. This choice aligns with the fact that the dataset comprises 7 distinct groups. Additionally, we have considered the variable *k* in the range of 3 to 10. The outcomes of these applications are illustrated in Fig 8. It was observed that the methods CRO-FADALRA, FB, FOM, ISFE, FOADARMAH, and FOADATH tend to classify entire classes as outliers, which may not be desirable in a practical scenario. On the contrary, OUG identifies only a few outliers within class 5, and RMAH fails to recognize any outliers.

In relation to the methods requiring an outlier rate, TRIMKMEANS does not identify the conspicuous outliers in class 5, whereas POND only detects these particular ones. The TRIM method categorizes an entire class as outliers, and HDR is the sole method that appears to detect not just the outliers in class 5, but also several more in other classes.

Notably, like the HDR method, the FAA+*k*NN approach stands out as it not only successfully detects the outliers in class 5 but also identifies additional outliers across other classes. This demonstrates its effectiveness in discerning subtle deviations within the data, thereby enhancing the robustness of outlier detection in this context.

#### 4.2.2 ECG200 data

**Fig 9:**
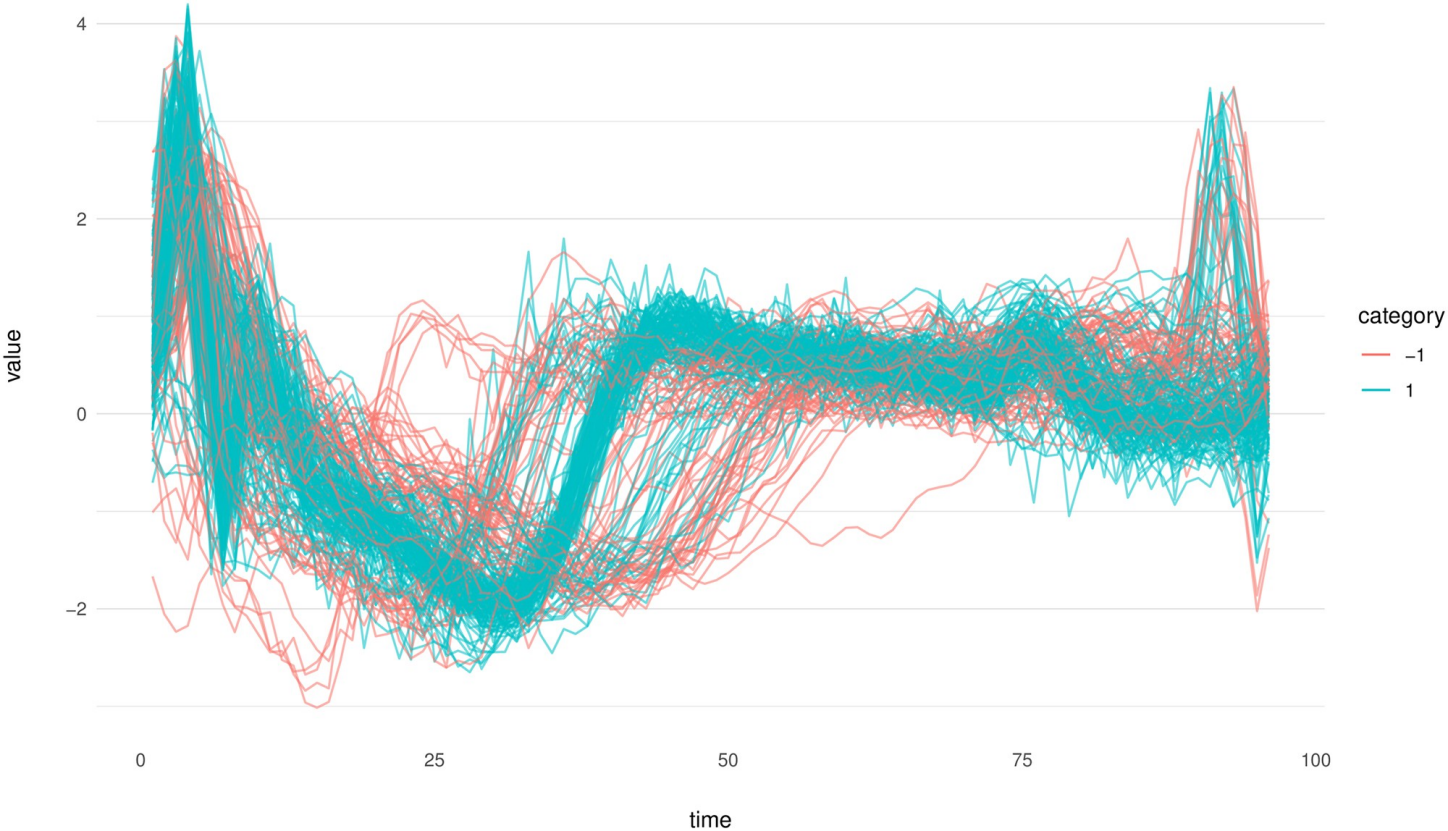
ECG200 dataset. Each line in this figure represents an individual electrocardiogram. The color coding of each line denotes the specific cluster to which the observation belongs.

The ECG200 dataset, introduced by [62], comprises 200 electrocardiograms and is accessible on the UCR Time Series Classification and Clustering website [63] and PhysioNet [64], and were collected in Beth Israel Hospital Arrythmia Laboratory in 1990 [65]. This dataset is divided into two distinct categories: one containing 133 electrocardiograms and the other comprising 67. Each electrocardiogram in the dataset is recorded at 96 equally spaced intervals. In Fig 9, the displayed electrocardiograms are illustrated.

In prior research, specifically in [51, 66], the detection of four outliers in the data set was noted. Consequently, this led to set an outlier rate of 0.02 (4/200 = 0.02). For TRIMKMEANS, 2 groups are considered. In our method, we set the parameters following the previously defined procedure. The RSS plot was a critical tool in this process, guiding our decision to select *p*_1_ = 3 and *p*_2_ = 8 as parameters. Furthermore, with the objective of identifying a small cluster comprising four outliers, we defined the parameter *k* to range between 5 and 50.

**Fig 10:**
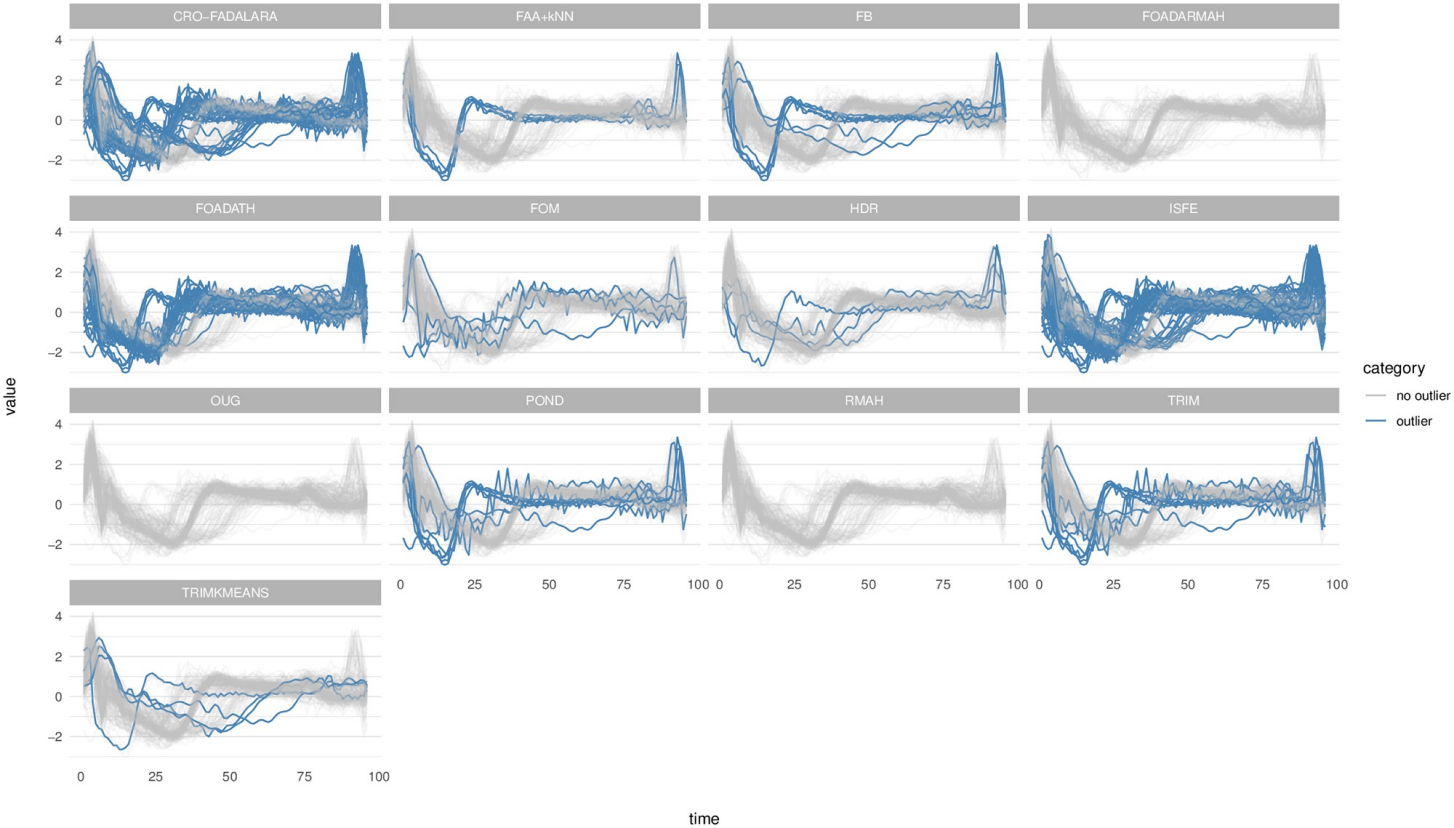
Outliers detected in the ECG200 data set for the different methods.

Fig 10 provides a comprehensive visual representation of all outliers identified by various methods. A key observation from this figure is that only our proposed method successfully detects the outliers that are aligned with the findings presented by [51, 66].

#### 4.2.3 Anomaly detection in image textures

**Fig 11:**
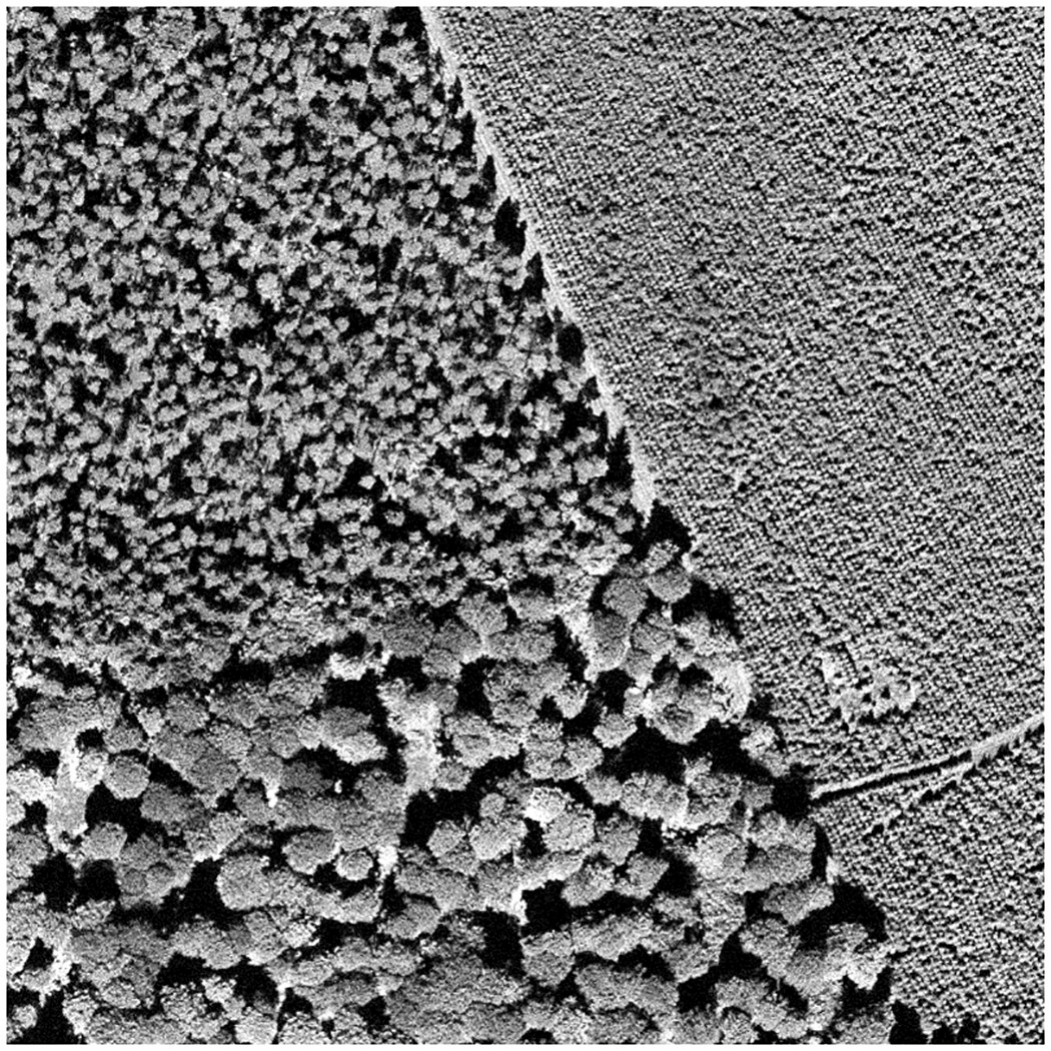
Image of a natural landscape with an anomaly, a small area without trees.

Detecting outliers in textured images poses a significant challenge due to the inherent complexity and variability of textures, especially in natural settings such as forests. An illustrative example of this challenge is presented in Fig 11, which displays an image of forest stands characterized by highly variable textures, complicating the processing task. This specific image has been previously analyzed by [67].

**Fig 12:**
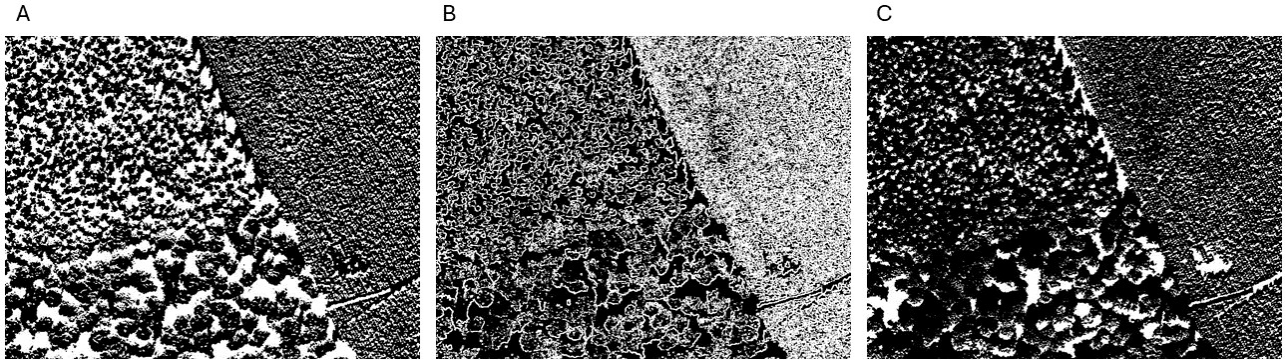
Binary images resulting from mathematical morphology transformations of Fig 11. A: it shows the extended-minima transform; B: it is a thresholded gradient; C: it shows a thresholded of a filling holes operation based on morphological reconstruction.

**Fig 13:**
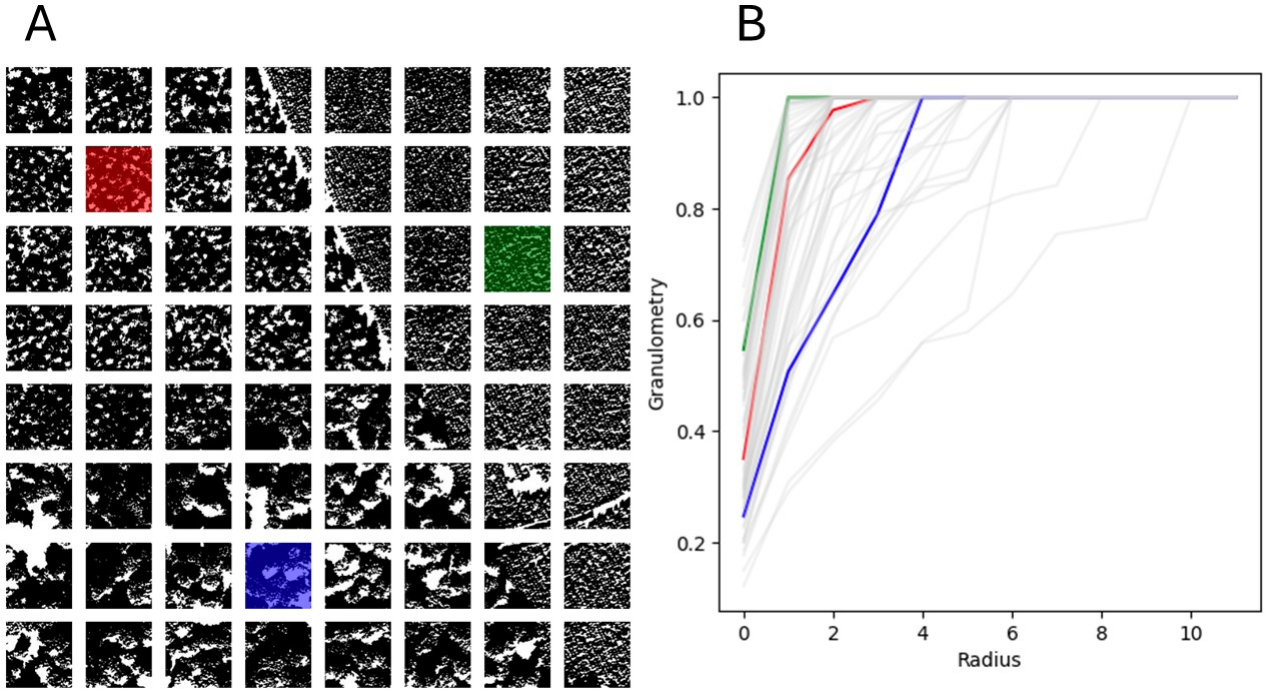
Granulometries resulting from a binary image transformation of Fig 11. A: it shows the image in tiles of 100 × 100. B: it shows the granulometry curves associated with each tile.

The image underwent three mathematical morphology transformations as outlined by [68], with the transformed states depicted in Fig 12. Subsequently, following the approach of [67], local granulometries were computed within systematically selected 100 × 100 windows, the regions of which are exhibited in Fig 13. This methodology for texture description has been validated as effective by both [67, 69], for example.

**Fig 14:**
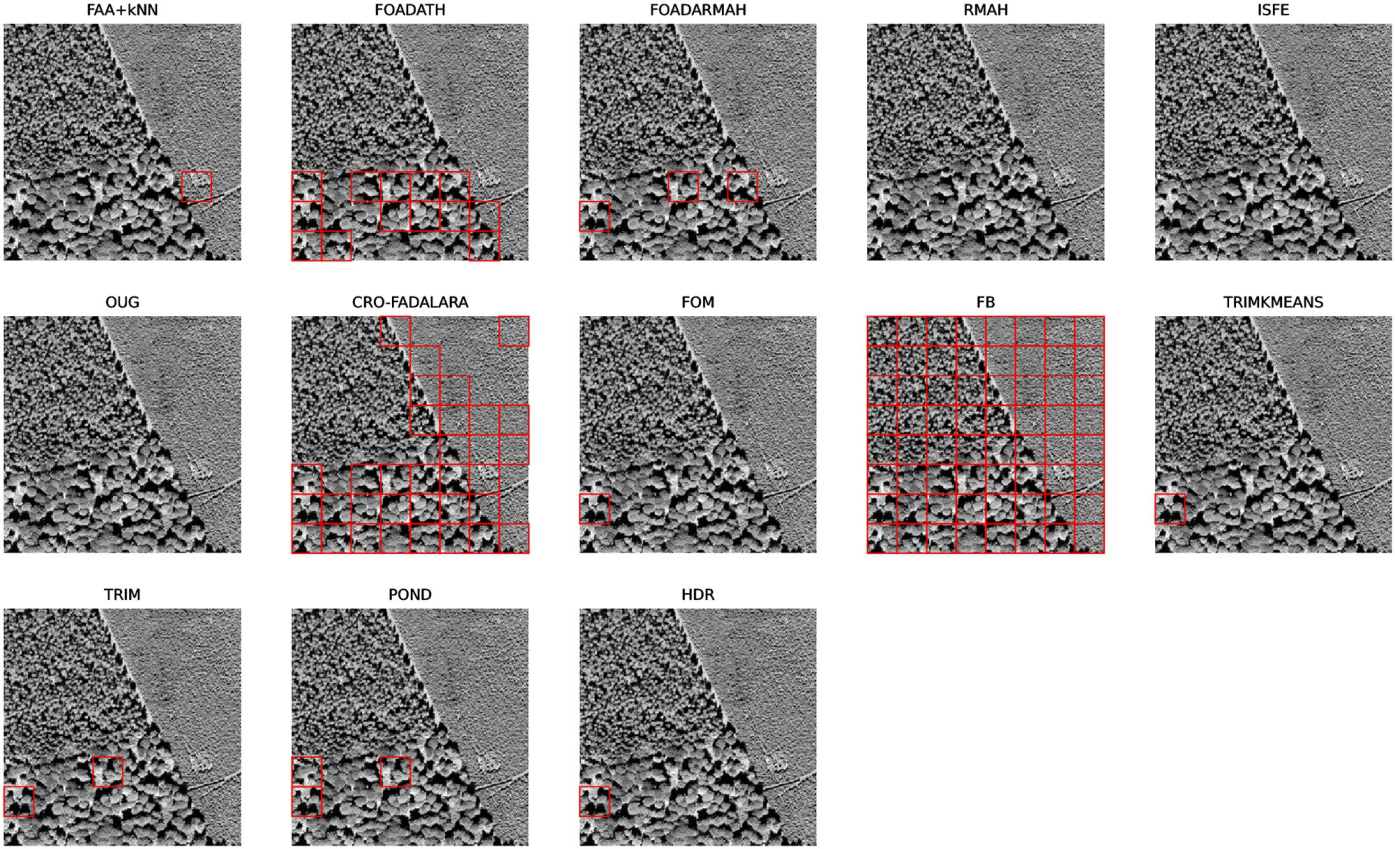
Anomalies detected by each method, shown in red.

For the analysis of these tri-variate functional data (the granulometries of the three transformations), methods described previously were employed. As we were looking for only one outlier, we set the outlier rate of 0.015 (1/64 ≃ 0.015). For TRIMKMEANS, 3 groups are considered. Given the modest sample size of 64, the parameters were set to *p*_1_ = 3 (there are three different sets of textures), *p*_2_ = 4 and *k* ranging from 1 to 3 in order to detect lonely outliers. The anomalies identified through this process are detailed in Fig 14.

Our method distinguishes itself by being the sole technique that accurately detects the anomaly within the dataset. In contrast, the alternate methods either fail to identify the anomaly, leading to a false negative, or incorrectly flag non-anomalous data as outliers, resulting in false positives. This comparison underlines the robustness and reliability of our proposed method in isolating true anomalies in challenging datasets where texture plays a predominant role.

A reviewer raised the question of the performance obtained with state-of-the-art deep learning based methods for image anomaly detection. Note that unsupervised deep learning-based image anomaly detection are not truly unsupervised because normal samples feed the algorithms, i.e. normal samples have to be included in the training set [70]. Therefore, the information about what is a normal sample is provided to the algorithm. This is an important difference with our proposal, which is fully unsupervised. In order to make the comparison, we have considered as training set the original tiles in rows from 1 to 4 and from 7 to 8 in Fig 13A for Fig 11, i.e. a total of 48 tiles. Test set, which contains the anomalous tile, is composed by the tiles in rows 5 and 6, i.e. a total of 16 samples. We have applied two state-of-the art methods: FastFlow [71] and PatchCore [72] with the Matlab functions fastFlowAnomalyDetector and patchCoreAnomalyDetector with default parameters, respectively. According with the outlier scores returned, the anomalous tile is the third most normal tile of the 16 test tiles with FastFlow; while, it is the most normal with PatchCore. Therefore, performance of deep-learning-based methods is very low in this case. Note that the deep learning has a good performance with large sample data sets. However, accuracy decreases in the small sample size setting due to difficulties in training and over-fitting [73]. Furthermore, processing natural landscapes is a high challenge. Mixture of natural (real) textures are more difficult to process than industrial images, where deep-learning-based methods have good performance, due to their high natural variation.

#### 4.2.4 3D shape of the left and right hippocampi

We consider the 3D shape of the left and right hippocampi of 28 individuals from Structural Magnetic Resonance Imaging (sMRI) brain scans. Anonymized data are publicly available at [74]. These data were introduced by [75]. As detailed in [75], all experimental procedures complied with the guidelines that were approved by the ethical research committee at the Universitat Jaume I. Written informed consent was obtained from every individual or their appropriate proxy prior to participation, complying with existing Spanish legislation (Ley Orgánica 15/1999, de 13 de diciembre, de Protección de Datos de Carácter Personal, LOPD) granting the use of the data for research purposes. Data were collected from June 21st 2004 to October 19th 2005 by the Neurology Service at La Magdalena Hospital (Castelló, Spain) and the Neuropsychology Service at the Universitat Jaume I.

**Fig 15:**
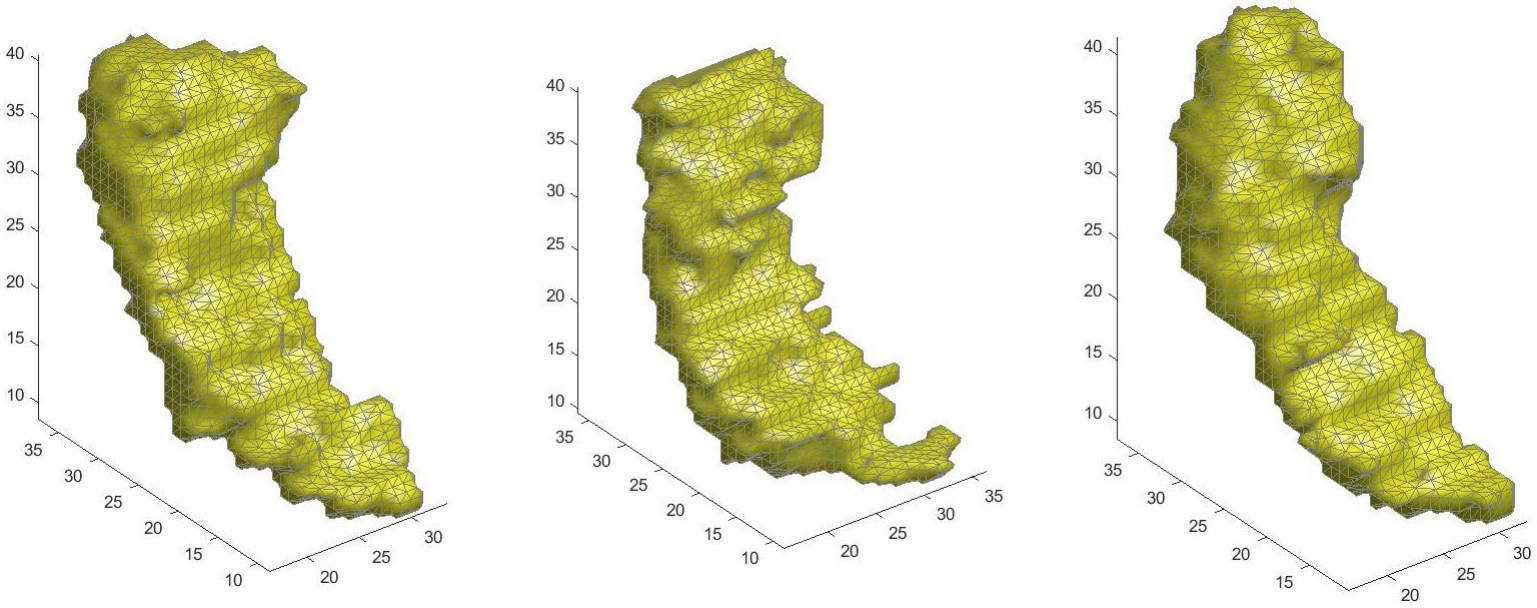
A left hippocampus for each group (from left to right): CN, MCI and AD.

They are divided into three groups: 12 cognitively normal (CN) subjects, 6 individuals with mild cognitive impairment (MCI) and 10 subjects with early Alzheimer’s Disease (AD). Each hippocampus is expressed as a three-vector-valued function in the unit sphere with spherical angles (*θ*, *φ*) by spherical harmonic representation. Therefore, we work with multivariate and multiargument functional data. As spherical harmonics are orthonormal, we can stack the vectors of SPHARM basis coefficients and work as in the multivariate case [12]. The data and their representation are described by [74, 76]. Fig 15 displays an example of one left hippocampus for each of the three groups.

We implement our methodology using *p*_1_ = 2 and *p*_2_ = 4, as indicated by the RSS plot. We set *k* to 2 given the small size of our sample. In this case, we analyze the outlier scores and their relationship with the degree of severity of the disease. The outlierness increases according to the degree of severity of the disease. An ordered logistic regression has been applied and for every one unit increase in outlier score the odds of being a more severe degree of AD increase 12.41-fold and 1.63-fold, for the left and right hippocampi, respectively. This difference between the two hippocampi is not surprising, since the shape of the left hippocampus is more discriminant than that of the right hippocampus, i.e., a better correct classification percentage is obtained using the information in the left hippocampus [76].

## 5 Conclusion

This study has introduced an innovative approach for the identification of outliers in functional data, which exhibits versatility across univariate, multivariate, and multi-dimensional functions. A significant advantage of our method over existing techniques is its ability to efficaciously detect outliers within clustered functional data. Through extensive testing on simulated and real datasets, we have demonstrated that our method not only excels when faced with clustered data scenarios but also maintains comparable efficacy to current methods in single-group contexts.

Looking ahead, there is potential for employing our outlier detection method as a preliminary step in clustering analyses to isolate and remove outliers, thereby refining the clustering process. Additionally, our approach holds promise for defect detection in textured images, which has implications for quality control across various industries, such as textiles and ceramics.

Future research directions could also include adapting our method for application to datasets comprising mixed types of data, encompassing functional, numerical, and categorical variables, as well as accommodating datasets with missing values adjusting the methodologies by [77], sparse functional data [78–80], directional data [81], shape datasets [82–84], ordinal [85] and binary [86] data sets, interval data [87], data cells for finding cellwise outliers [88], among others. These enhancements would further the applicability of our approach in a wider array of analytical scenarios, thereby broadening the impact and utility of our work in the field of data analysis. Another future research direction is to apply the proposal to anomaly detection on (multivariate) time series [89, 90] by transforming the long (multivariate) time series into a (multivariate) functional data set by dividing the time axis into intervals depending on the period, as made by [14], for instance. Finally, other implementations [91] for obtaining archetypes could be considered.
